# Bioactivities of Lyngbyabellins from Cyanobacteria of *Moorea* and *Okeania* Genera

**DOI:** 10.3390/molecules25173986

**Published:** 2020-09-01

**Authors:** Imam Fathoni, Julie G. Petitbois, Walied M. Alarif, Ahmed Abdel-Lateff, Sultan S. Al-Lihaibi, Erina Yoshimura, Yasuyuki Nogata, Charles S. Vairappan, Eti Nurwening Sholikhah, Tatsufumi Okino

**Affiliations:** 1 Graduate School of Environmental Science, Hokkaido University, Sapporo 060-0810, Japan; tonythoni@gmail.com (I.F.); juliepetitbois@ees.hokudai.ac.jp (J.G.P.); 2 Department of Marine Chemistry, Faculty of Marine Sciences, King Abdulaziz University, P.O. Box 80207, Jeddah 21589, Saudi Arabia; walied1737@yahoo.com (W.M.A.); sallihaibi@kau.edu.sa (S.S.A.-L.); 3 Department of Natural Products and Alternative Medicine, Faculty of Pharmacy, King Abdulaziz University, P.O. Box 80260, Jeddah 21589, Saudi Arabia; ahmedabdellateff@gmail.com; 4 Department of Pharmacognosy, Faculty of Pharmacy, Minia University, Minia 61519, Egypt; 5 CERES, Inc., 1-4-5 Midori, Abiko, Chiba 270-1153, Japan; erinamusi@yahoo.co.jp; 6 Environmental Science Research Laboratory, Central Research Institute of Electric Power Industry, Abiko 270-1194, Japan; noga@criepi.denken.or.jp; 7 Institute for Tropical Biology and Conservation, Universiti Malaysia Sabah, Kota Kinabalu 88450, Sabah, Malaysia; csv@ums.edu.my; 8 Faculty of Medicine, Public Health, and Nursing, Universitas Gadjah Mada, Yogyakarta 55281, Indonesia; etinurweningsholikhah@ugm.ac.id; 9 Faculty of Environmental Earth Science, Hokkaido University, Sapporo 060-0810, Japan

**Keywords:** cyanobacteria, antiplasmodial, cytotoxic, antifouling, *Moorea*, *Okeania*

## Abstract

Cyanobacteria are reported as rich sources of secondary metabolites that provide biological activities such as enzyme inhibition and cytotoxicity. Ten depsipeptide derivatives (lyngbyabellins) were isolated from a Malaysian *Moorea bouillonii* and a Red Sea *Okeania* sp.: lyngbyabellins G (**1**), O (**2**), P (**3**), H (**4**), A (**7**), 27-deoxylyngbyabellin A (**5**), and homohydroxydolabellin (**6**). This study indicated that lyngbyabellins displayed cytotoxicity, antimalarial, and antifouling activities. The isolated compounds were tested for cytotoxic effect against human breast cancer cells (MCF7), for antifouling activity against *Amphibalanus amphitrite* barnacle larvae, and for antiplasmodial effect towards *Plasmodium falciparum*. Lyngbyabellins A and G displayed potent antiplasmodial effect against *Plasmodium*, whereas homohydroxydolabellin showed moderate effect. For antifouling activity, the side chain decreases the activity slightly, but the essential feature is the acyclic structure. As previously reported, the acyclic lyngbyabellins are less cytotoxic than the corresponding cyclic ones, and the side chain increases cytotoxicity. This study revealed that lyngbyabellins, despite being cytotoxic agents as previously reported, also exhibit antimalarial and antifouling activities. The unique chemical structures and functionalities of lyngbyabellin play an essential role in their biological activities.

## 1. Introduction

Cyanobacteria are often associated with harmful algal blooms as well as their ability to produce diverse structurally and biologically active secondary metabolites, including enzyme inhibitors, antifouling, anticancer, antibiotic, or antiparasitic compounds [1,2]. Marine organisms have traditionally attracted the attention of many scientists in order to find potential active compounds, develop effective treatments of neglected diseases such as malaria, where the evolution of resistance of the parasite to current malaria drugs requires the discovery of new compounds with novel mechanisms of action [3], or deal with environmental problems such as those induced by biofouling by more eco-friendly coatings [4,5,6,7]. Valorizing them could lead to the potential isolation of active compounds. Moreover, cyanobacteria can be cultivated in photobioreactors with precisely controlled culture conditions producing active compounds [8].

As part of an effort to identify antiplasmodial and antifouling metabolites, two cyanobacteria from two geographically distinct environments were studied: a Malaysian *Moorea bouillonii* and a Red Sea *Okeania* sp. *Moorea* and *Okeania* genera were delineated from the *Lyngbya* genus [9,10]. As phylogenetically close genera, they produce similar compounds. In this study, the lyngbyabellin family depsipeptides were isolated from *Moorea bouillonii* and *Okeania* sp. These kinds of compounds were initially found in the sea hare *Dolabella auricularia* after being identified from cyanobacterial origins as part of the sea hare diet [11,12]. These depsipeptides are characterized by two thiazole rings and an unusual *gem-*dichloro group. Lyngbyabellins G, O, and P have recently been shown to inhibit the settlement of *Amphibalanus amphitrite* barnacle larvae. At the same time, such compounds are reported as antimalarial agents [13]. The number of marine natural products which were tested against *Plasmodium* is limited but increasing. Several compounds show promising activities as future antimalarial agents [5] (Table 1). Dolastatin 10, a peptide microtubule inhibitor isolated from *D. auricularia*, is considered as the most potent antimalarial compound of cyanobacteria (IC50 = 0.1 nM). Despite its low selectivity index against various cancer cell lines (≤1), dolastatin 10 is one of the most potent anticancer drugs [14]. Other compounds showing promising activity against different types and stages of malaria were also isolated from cyanobacteria such as dolastatin 15, gallinamide A, hoshinoamide A, carmabin A, dragomabin, venturamide A, and companeramide A [15,16,17,18,19,20]. *Moorea* and *Okeania* genera already provided several antimalarial molecules, including mabunamide, ikoamide, kakeromamide B, ulongamide A, lyngbyabellin A, and bastimolides A and B [13,21,22,23,24].

## 2. Results and Discussion

### 2.1. Isolation and Characterization of Compounds

**Figure 1:**
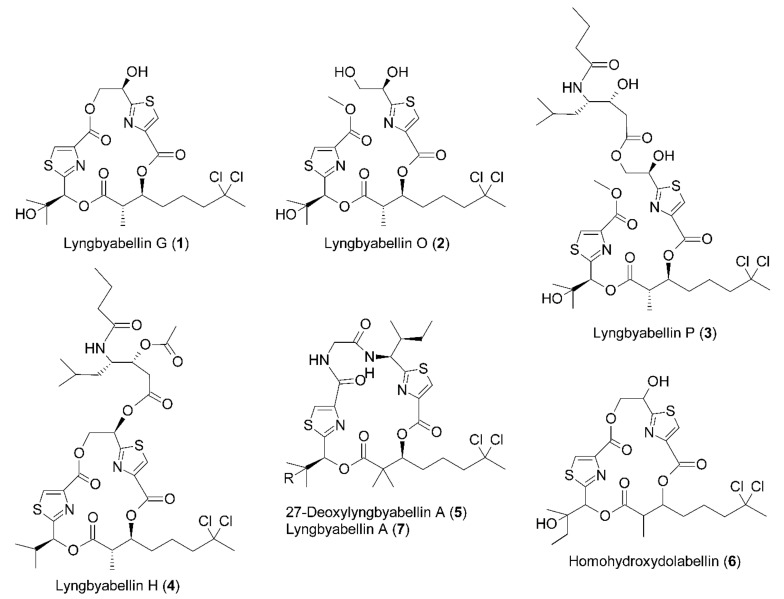
Structure of depsipeptides isolated for this study: lyngbyabellin G (**1**), lyngbyabellin O (**2**), lyngbyabellin P (**3**), lyngbyabellin H (**4**), 27-deoxylyngbyabellin A, R = H (**5**), homohydroxydolabellin (**6**), and lyngbyabellin A, R = OH (**7**). Compounds **1**–**5** are from *Okeania* sp. and **6**–**7** from *M. bouillonii*.

The MeOH extracts of *Okeania* sp. and *M. bouillonii* were partitioned between EtOAc and H_2_O. The EtOAc fraction was separated into nine subfractions via normal phase silica gel column chromatography. The fractions obtained were subjected to Liquid Chromatography-Electrospray Ionization- Mass Spectrometry (LC-ESI-MS) analysis. All isolated compounds (Figure 1) provided 9:6:1 isotope peaks, revealing the presence of two chlorine atoms. Compounds **1**–**3** were previously isolated from this *Okeania* sp. (Saudi Arabia) extract [25], together with compounds **4** and **5**. Compounds **6** and **7** were isolated from the other cyanobacterium, *M. bouillonii* (Malaysia). The identities of the additional compounds **4**–**7** were confirmed by comparison with the chemical shifts reported in the literature [12,26,27,28].

### 2.2. Antifouling Activity

A previous study on the antifouling activity of **1**–**3** showed that these compounds inhibit the settlement of *A. amphitrite* barnacle larvae [25]. Indeed, **1** showed low activity (EC_50_ 4.41 µg/mL), while **2** and **3** exhibited potent activity (EC_50_ 0.24 and 0.62 μg/mL, respectively) after 48 h of exposure. In the current study, **4** was inactive at 10 µg/mL. These results suggest that the larvae are more sensitive to the acyclic structure of **2** and **3** than the cyclic structure of **1** and **4**, and that the presence of a side chain slightly decreases the activity (**1** and **2** being more active than **3** and **4**, respectively). Compound **5** was the most potent (EC_50_ 0.09 µg/mL), but its activity was related to its high toxicity (almost all the larvae were dead when exposed to 1 µg/mL), probably due to the presence of two extra lactams, which is the main difference compared with the other tested lyngbyabellins. As for all the compounds, the activity remained after 120 h of exposure.

### 2.3. Cytotoxicity

The cytotoxicity of **1**–**3** with respect to MCF7 breast cancer cells was already reported in a previous study, whose IC_50_ were 120, >160, and 9 µM, respectively [25]. In this study, the IC_50_ values of **4** and **5** were 0.07 and 0.31 µM, respectively. Compound **6** showed non-cytotoxicity with respect to MCF7 breast cancer cells. These differences in cytotoxicity were attributed to the dissimilarities in their structures. Cyclic lyngbyabellin with a side chain (**4**) is the most active, while an acyclic form or the lack of a side chain decreases cytotoxicity [12].

### 2.4. Antiplasmodial Activity

Compound **1** isolated from *Okeania* sp., and compounds **6** and **7** from *M. bouillonii* were tested against the malaria parasite (*P. falciparum*). Compounds **1** and **7** were active towards the malaria parasite (IC_50_ 1.1 and 0.3 μM, respectively), while **6** was moderately active (IC_50_ 6.4 μM). The antiplasmodial activity of compound **7** is less potent than what was reported by Sweeney-Jones et al. [13]. This is presumably because compound **7** exhibits a better inhibition towards the liver stage of *Plasmodium* compared to the intraerythrocytic stage used in this research. The assay was repeated three times in the concentration range of 0.05–10 (μg/mL). It is noteworthy that the mechanisms of antiplasmodial compounds isolated from *M. bouillonii* impeding the growth of *Plasmodium* are still unclear. According to Peatey et al. [29], until recently, most of the antimalarial drugs developed only blocked the asexual stage of parasites. However, as **1** and **7** were reported as cytotoxic towards cancer cells, we hypothesized that they act in the same way on erythrocytes, which distorts the results of the antimalarial assay.

## 3. Materials and Methods

### 3.1. Cyanobacteria Collection

*Okeania* sp. was collected on the Algetah Alkabira reef near Jeddah, Saudi Arabia (N 21°41′23.98″; E 39°00′52.94″) in April 2015, while *Moorea bouillonii* was collected in March 2014 and September 2016 at Sabah, Malaysia. Foreign particles were removed by hand and both cyanobacterial extracts were squeezed by hand to remove any seawater before being stored in MeOH for transportation.

### 3.2. Identification of Cyanobacteria

A small portion of the materials was also preserved in 10 mL of RNAlater solution for genetic analysis. Voucher specimens of *Okeania* sp. (S1501) and *M. bouillonii* (M1415) preserved in RNAlater were deposited at Hokkaido University, Japan.

### 3.3. Extraction and Isolation of Compounds

Both extracts were homogenized and extracted three times with MeOH. The dried MeOH extracts were then partitioned between EtOAc and H_2_O (1:1, *v/v*). The isolation of lyngbyabellins G (**1**), O (**2**), and P (**3**) from this *Okeania* sp. extract was previously reported [25]. Briefly, the dried EtOAc fraction was purified by NP-column chromatography over silica gel. A stepwise gradient composed of hexane and EtOAc, followed by EtOAc and MeOH, was used to elute the lyngbyabellins. The 75:25 EtOAc/MeOH fraction was fractionated by RP-HPLC over a Cosmosil Cholester column (10 × 250 mm, 5 μm; gradient 0–50 min, 75–90% MeCN containing 0.05% (*v/v*) trifluoroacetic acid (TFA), UV detection at 210 nm, and flow rate of 3 mL/min) to obtain **1**, **2**, and **3** (3.0, 1.8, and 2.0 mg, respectively). Lyngbyabellins H (**4**) and 27-deoxylyngbyabellin A (**5**) were isolated from the 4:6 hexane/EtOAc. This fraction was purified by the same HPLC system (gradient 0–60 min, 50–70% MeCN). The subfraction was eluted at t_R_ 46.1–46.5 min; 3.0 mg was further purified by RP-HPLC (gradient 0–60 min: 50–70% MeCN) to provide **5** (t_R_ 36.3 min, 1.6 mg) and **4** (t_R_ 38.9 min, 1.1 mg).

Homohydroxydolabellin (**6**), a dichlorinated compound, was isolated from fraction EtOAc 100% and EtOAc/MeOH 75:25 of *M. bouillonii.* The compound produced [M + H]^+^ peaks at *m/z* 641.1089 (t_R_ 5.4–5.8 min) and the yield was 1.1 mg. The compound was further purified by HPLC (isocratic 40% MeCN, flow rate 1 mL/min, column Cosmosil Cholester 4.6 × 250 mm, wavelength 210 nm). The final amount of the compound was 0.6 mg. Lyngbyabellin A (**7**) was also isolated from this fraction, similar to the method previously described (t_R_ 13.3–14.6 min, 0.5 mg). The identity of the compounds was confirmed by a combination of ^1^H NMR spectroscopy (CDCl_3_, 400 MHz) and MS analysis (Supplementary Materials: Tables S1–S4 and Figures S5–S8). In our previous study, the identity of **1** was confirmed by comparison of ^1^H and ^13^C NMR data with the literature (the largest difference in ^13^C chemical shifts was 0.0028 ppm), together with similar specific rotation ([α]^26^_D_ −28.6 for **1** and −26 reported for lyngbyabellin G) [12,25]. As compounds **1**−**5** were isolated from the same organism, their configurations were assumed to be identical.

### 3.4. Antifouling Assay on A. amphitrite

The antifouling activity of the compounds was determined according to a procedure reported previously [25]. Briefly, the newly developed cyprids of *A. amphitrite* were tested against several different concentrations of the isolated compounds (**1**–**5**) (0.03–10 μg/mL) in triplicate (n = 3). The assays were conducted using 24-well polystyrene culture plates, with each well containing 2 mL seawater and six cyprids. After 48 h exposure to darkness, the numbers of cyprids that settled or did not settle were counted under a light microscope. The total number of dead cyprids was counted after 120 h of exposure. Antifouling activity was expressed as an EC_50_ according to Probit analysis. CuSO_4_ was used as a positive control and 80% filtered (0.20 µm of pore filter) natural seawater (diluted with deionized water) as a negative control.

### 3.5. Cytotoxicity Assay on MCF7 Breast Cancer Cells

The cytotoxicity of the compounds was determined according to a procedure reported previously [30]. MCF7 breast cancer cells were maintained in RPMI-1640 medium with 10% fetal bovine serum (FBS). The cells were plated into 96-well plates with a density of 1.0 × 10^4^ cells per well. After 24 h incubation, the cells were exposed to different concentrations of compounds, with cisplatin as a positive control and with 1% filtered ethanol as a negative control. The MTT assay was conducted after 72 h of incubation at 37 °C and 5% CO_2_.

### 3.6. Antiplasmodial Assay

The Giemsa staining method previously described by Aydin-Schmidt [31] was used to evaluate the ability of compounds to inhibit the malaria parasite. A synchronized culture of *Plasmodium falciparum* strain FCR-3, the late-ring stage, was used (parasitemia 2%) for this assay [31]. A series of compound concentrations were applied in 96-well microplates and incubated for 72 h at 37 °C in a reduced oxygen atmosphere. Compounds **1**, **6**, and **7** were dissolved in DMSO and tested in triplicate at three separate times. Chloroquine was used as a positive control and cultured medium with 1% DMSO (final concentration of solvent in all compound tested) as a negative control. Each assay was conducted in triplicate (n = 3). After 72 h, the parasitic level was evaluated by Giemsa and light microscopy. The inhibition level of compounds against *Plasmodium* was calculated using the formula previously reported [31,32].

## 4. Conclusions

Two cyanobacteria from two distinct environments but with genetic similarities provided several derivatives of the lyngbyabellin family: *M. bouillonii* (Malaysia) and *Okeania* sp. (Saudi Arabia). These compounds are mainly known to inhibit several cancer cell lines proliferation (Table 2). We increased the scope of the bioactivities of lyngbyabellins by studying their antimalarial and antifouling activities (Table 2).

This study added more evidence that their structure affects their activity. Indeed, as previously reported, cyclic structures with a side chain are the most cytotoxic [12,25]. Lyngbyabellins are known as actin-disrupting agents, and therefore, act on the cellular microfilaments network [12,13,26,27]. This disruption leads to the impossibility of cells to enter mitosis, giving abnormal cells with two nuclei. As a result, apoptosis occurs. Erythrocytes used in antiplasmodial assays are likely affected in the same way. Thus, cytotoxicity is considered to provide the antimalarial activity of lyngbyabellins, which precludes the direct action on the parasite (non-specific mechanisms). Homohydroxydolabellin (**6**) showed non-cytotoxicity towards MCF7 cells (>50 μg/mL) but moderate antiplasmodial activity, making it a potent compound to treat malaria. Further assays on the blood cells only will be performed because the challenge of developing a new treatment is finding a substance that is effective against malaria, safe to humans (not destroying erythrocytes), cheap, and easy to be produced. 

On the contrary, lyngbyabellins possessing an acyclic structure without a side chain are the most active towards the settlement of barnacle larvae. 27-deoxylyngbyabellin A (**5**) is the only toxic compound and the only one to have two extra lactams. Its toxicity is presumably due to this specificity. The flexibility of the acyclic structures may help interactions with a common target, while the addition of a side chain decreases them. The structures of these depsipeptides are characterized by two thiazole rings and an unusual *gem*-dichloro group. Currently, no structure–activity relationship antifouling studies have been conducted on compounds containing these features. Synthesis of more analogs for structure–activity relationship studies would improve our knowledge on their activities.

## Figures and Tables

**Table 1:** Structural diversity and antimalarial potential of secondary metabolites derived from cyanobacteria.

Compounds	Sources	Structural Class	IC_50_ (µM)	Assay Method	References
Dolastatin 10	*Dolabella auricularia*	peptide	0.0001	pLDH	[15]
Dolastatin 15	*Symploca* sp.	peptide	0.0024	pLDH	[15]
Gallinamide A	*Schizotrix* sp.	depsipeptide	8.4	Pico-Green	[16]
Hoshinoamide A	*Caldora penicillate*	lipopeptides	5.2	pLDH	[17]
Carmabin A	*Lyngbya. majuscula*	lipopeptides	4.3	Pico-Green	[18]
Dragomabin	*L. majuscula*	lipopeptides	6.0	Pico-Green	[18]
Venturamide A	*Oscillatoria* sp.	hexapeptides	8.2	Pico-Green	[19]
Companeramide A	*Leptolyngbya*	depsipeptide	5.7	Sybr Green	[20]
Mabunamide	*Okeania* sp.	lipopeptides	1.4	pLDH	[21]
Ikoamide	*Okeania* sp.	lipopeptides	0.14	pLDH	[22]
Kakeromamide B	*Moorea producens*	pentapeptides	8.9	Sybr Green	[13]
Ulongamide A	*M. producens*	depsipeptides	0.99	Sybr Green	[13]
Lyngbyabellin A	*M. producens*	depsipeptides	0.0015	Sybr Green	[13]
Bastimolide A	*Okeania hirsuta*	polyhydroxy macrolide	0.27	Sybr Green	[23]
Bastimolide B	*O. hirsuta*	macrolide	5.7	Pico-Green	[24]

**Table 2:** Diversity of biological activities reported for Lyngbyabellins in the literature from 2000 to 2020.

Compounds	Bioactivities	IC_50_ (µM)	EC_50_(µg/mL)	Cells Lines/Organisms	References
Lyngbyabellin A	Antimalarial, anticancer	0.021–0.345		HT29, HeLA, KB, LoVo	[13,26,27], current study
		0.0015		*P. falciparum* (liver-stage)	
		0.3		*P.falciparum* (intraerythrocytic stage)	
27-deoxylyngbyabellin A	Antifouling, anticancer	0.0073–0.31		HT29, HeLA, MCF7	[27], current study
			0.09 (but toxic)	*A.amphitrite* barnacle cypris	
Lyngbyabellin B	Anticancer	0.1–1.1		HT29, HeLA, CA46, PtK2	[27,33]
Lyngbyabellin C	Anticancer	2.1–5.3		KB, LoVo	[28]
Lyngbyabellin D	Anticancer	0.1		KB	[34]
Lyngbyabellin E	Anticancer	0.4–1.2		H460, Neuro-2a	[12]
Lyngbyabellin F	Anticancer	1.0–1.8		H460, Neuro-2a	[12]
Lyngbyabellin G	Antimalarial, anticancer, antifouling	2.2–120		H460, Neuro-2a, MCF7	[12,25], current study
			4.41	*A.amphitrite* barnacle cypris	
				*P.falciparum* (intraerythrocytic stage)	
Lyngbyabellin H	Anticancer	0.07–1.4		H460, Neuro-2a, MCF7	[12], current study
			Inactive	*A.amphitrite* barnacle cypris	[12]
Lyngbyabellin I	Anticancer	0.7–1.0		H460, Neuro-2a	[12]
Lyngbyabellin J	Anticancer	0.041–0.054		HT29, HeLA	[12]
Lyngbyabellin K		Inactive		H460	[35]
Lyngbyabellin L		Inactive		H460	[35]
Lyngbyabellin M		Inactive		H460	[35]
Lyngbyabellin N	Anticancer	0.0048–1.8		H460, HCT116	[35]
Lyngbyabellin O	Antifouling	Inactive		MCF7	[25]
			0.24	*A.amphitrite* barnacle cypris	
Lyngbyabellin P	Anticancer, antifouling	9.0		MCF7	[25]
			0.62	*A.amphitrite* barnacle cypris	

## Supplementary Materials

The following are available: Tables S1–S4; tables comparison of chemical shifts of the compounds isolated in the current study with the literature values. Figures S5–S8; HPLC and LC/MS spectra of the isolation of lyngbyabellin H, 27-hydroxylyngbyabellin A, and homohydroxydolabellin.
