# Supplementary material for: Bioactivities of Lyngbyabellins from Cyanobacteria of Moorea and Okeania Genera
Source: Molecules. 2020 Sep 1;25(17):3986. doi: 10.3390/molecules25173986 (PMC7504728; doi:10.3390/molecules25173986)
Supplement: Supplementary file 1 [file molecules-25-03986-s001.pdf]

Supporting Information

Bioactivities of Lyngbyabellins from Cyanobacteria of *Moorea* and *Okeania* Genera

Imam Fathoni [1], Julie G. Petitbois [1], Walied M. Alarif [2], Ahmed Abdel-Lateff [3,4], Sultan S. Al-Lihaibi [2], Erina Yoshimura [5], Yasuyuki Nogata [6], Charles S. Vairappan [7], Eti Nurwening Sholikhah [8] and Tatsufumi Okino [1,9,*]

[1] Graduate School of Environmental Science, Hokkaido University, Sapporo, Japan. tonythoni@gmail.com, juliepetitbois@ees.hokudai.ac.jp, okino@ees.hokudai.ac.jp

[2] Department of Marine Chemistry, Faculty of Marine Sciences, King Abdulaziz University, P.O. Box 80207, Jeddah 21589, Saudi Arabia. walied1737@yahoo.com, sallihaibi@kau.edu.sa

[3] Department of Natural Products and Alternative Medicine, Faculty of Pharmacy, King Abdulaziz University, P.O. Box 80260, Jeddah 21589, Saudi Arabia. abdellatteff@kau.edu.sa

[4] Department of Pharmacognosy, Faculty of Pharmacy, Minia University, Minia 61519, Egypt. ahmedabdellateff@gmail.com

[5] CERES, Inc., 1-4-5 Midori, Abiko, Chiba 270-1153, Japan. erinamusi@yahoo.co.jp

[6] Environmental Science Research Laboratory, Central Research Institute of Electric Power Industry, Abiko 270-1194, Japan. noga@criepi.denken.or.jp

[7] Institute for Tropical Biology and Conservation, Universiti Malaysia Sabah, Kota Kinabalu 88450, Sabah, Malaysia. csv@ums.edu.my

[8] Faculty of Medicine, Public Health, and Nursing, Universitas Gadjah Mada, Yogyakarta, Indonesia.etinurweningsholikhah@ugm.ac.id

[9] Faculty of Environmental Earth Science, Hokkaido University, Sapporo, Japan. okino@ees.hokudai.ac.jp

* Correspondence: okino@ees.hokudai.ac.jp; Tel.: +81-11-706-4519

CONTENTS

S1. Comparison of the ^{1}H and ^{13}C chemical shifts of the isolated lyngbyabellin H with the literature values.. 3

S2. Comparison of the ^{1}H and ^{13}C chemical shifts of the isolated 27-deoxylyngbyabellin A with the literature values. 4

S3. Comparison of the ^{1}H chemical shifts of the isolated lyngbyabellin A with the literature values. ... 5

S4. Comparison of the ^{1}H chemical shifts of the isolated homohydroxydolabellin with the literature values.. 6

S5. RP-HPLC spectrum for the isolation of 27-deoxylyngbyabellin A and lyngbyabellin H. 7

S6. LC/MS spectra for 27-deoxylyngbyabellin A and lyngbyabellin H. 7

S7. RP-HPLC spectrum for the isolation of homohydroxydolabellin. 8

S8. LC/MS spectra for homohydroxydolabellin. 8

S1. Comparison of the ^{1}H and ^{13}C chemical shifts of the isolated lyngbyabellin H with the literature values[1].

	Literature		Current study		Differences	
**Position**	δ_C	δ_H (J in Hz)	δ_C	δ_H (J in Hz)	C	H
**1**	174.2		174		0.0011	
**2**	43.5	3.02	43.2	3.09	0.0069	-0.0231
**3**	74.9	5.35	74.8	5.35	0.0013	0
**4**	31.1	1.79, 1.75	30.8	NO	0.0096	NA
**5**	20.8	1.75, 1.73	20.6	NO	0.0096	NA
**6**	49.3	2.26, 2.15	49.1	2.27, 2.15	0.0041	-0.0044, 0
**7**	90.4		90.2		0.0022	
**8**	37.6	2.1	37.4	2.1	0.0053	0
**9**	15.1	1.27	14.9	1.27	0.0132	0
**10**	159.8		159.7		0.0006	
**11**	146.6		146.6		0	
**12**	128.6	8.09	128.4	8.1	0.0016	-0.0012
**13**	165.5		164.9		0.0036	
**14**	70.4	6.39	70	6.38	0.0057	0.0016
**14-OH**						
**15**	64.2	4.77	64.3	4.76	-0.0016	0.0021
**16**	160.7		160.5		0.0012	
**17**	146.3		145.9		0.0027	
**18**	129.2	8.2	129	8.19	0.0015	0.0012
**19**	168.1		168.4		-0.0018	
**20**	77.3	5.52	77.3	5.53	0	-0.0018
**21**	32.5	2.47	32.7	2.45	-0.0062	0.0081
**22**	19.2	0.92	19.2	0.93	0	-0.0109
**23**	18.5	1.06	18.4	1.06	0.0054	0
**24**	169.2		169.1		0.0006	
**25**	36.5	2.82	36.1	2.82	0.0110	0
		2.74		2.74		0
**26**	72.6	5.18	72.3	5.18	0.0041	0
**27**	49.3	4.32	49	4.33	0.0061	-0.0023
**27-NH**		5.86		6.11		-0.0427
**28**	40	1.3	39.9	1.28	0.0025	0.0154
**29**	25	1.62	24.8	NO	0.0080	NA
**30**	21.8	0.88	21.6	0.89	0.0092	-0.0114
**31**	23.7	0.94	23.4	0.94	0.0127	0
**32**	173.1		173		0.0006	
**33**	38.9	2.14	38.7	2.15	0.0051	-0.0047
**34**	19.4	1.62	19.2	NO	0.0103	NA
**35**	13.9	0.91	13.7	0.92	0.0144	-0.0110
**36**	170.6		170.4		0.0012	
**37**	21.2	2.05	21	2.04	0.0094	0.0049

[1] Han, B.; McPhail, K.L.; Gross, H.; Goeger, D.E.; Mooberry, S.L.; Gerwick, W.H. Isolation and structure of five lyngbyabellin derivatives from a Papua New Guinea collection of the marine cyanobacterium *Lyngbya majuscula*. *Tetrahedron* **2005**, *61*, 11723-11729. DOI: 10.1016/j.tet.2005.09.036

NO: not observed. NA: not available.

S2. Comparison of the ^{1}H and ^{13}C chemical shifts of the isolated 27-deoxylyngbyabellin A with the literature values[2].

	Literature		Current study		Differences	
**Position**	δ_C	δ_H (*J* in Hz)	δ_C	δ_H (*J* in Hz)	C	H
**1**	173.1		173.5		-0.0023	
**2**	46.5		46.5		0	
**3**	78.2	5.3	78.2	5.31	0	-0.0019
**4**	29.5	1.71, 1,40	29.4	NO, 1.42	0.0034	NA, -0.0143
**5**	22.3	1.6	22.4	NO	-0.0045	NA
**6**	49.3	2.22, 2.01	49.3	2.24, 2.06	0	-0.0090, -0.0249
**7**	90		90		0	
**8**	37.1	2.05	37.1	2.06	0	-0.0049
**9**	24.1	1.28	24.1	1.26	0	0.0156
**10**	20.2	1.34	20.3	1.35	-0.0050	-0.0075
**11**	160.5		160.9		-0.0025	
**12**	146.9		146.8		0.0007	
**13**	127.5	8.06	127.8	8.09	-0.002353	-0.0037
**14**	168.3		168.5		-0.0012	
**15**	55.1	5.23	55.1	5.23	0	0
**NH-15**		7.26		7.4		-0.0193
**16**	40.1	1.96	40	1.97	0.0025	-0.0051
**17**	25.4	1.50, 1.13	25.5	NO, 1.12	-0.0039	NA, 0.0088
**18**	11.2	0.92	11.3	0.93	-0.0089	-0.0109
**19**	14.9	0.77	14.9	0.77	0	0
**20**	168.3		168.5		-0.0012	
**21**	42.9	4.61	43	4.6	-0.002331	0.0022
**NH-21**		7.97		8.01		-0.0050
**22**	160.6		161.4		-0.0050	
**23**	148.7		148.7		0	
**24**	124.7	8.2	124.9	8.2	-0.0016	0
**25**	168.5		168.2		0.0018	
**26**	76.7	5.95	76.5	5.84	0.0026	0.0185
**27**	32.9	2.34	32.8	2.34	0.0030	0
**28**	18.7	0.95	18.9	0.95	-0.0107	0
**29**	18.6	1.1	18.7	1.1	-0.0054	0

[2] Matthew, S.; Salvador, L.A.; Schupp, P.J.; Paul, V.J.; Luesch, V.J .Cytotoxic halogenated macrolides and modified peptides from the apratoxin-producing marine cyanobacterium *Lyngbya bouillonii* from Guam. *J. Nat. Prod*. **2010**, 73, 1544-1552. DOI: 10.1021/np1004032

NO: not observed. NA: not available.

S3. Comparison of the ^{1}H chemical shifts of the isolated lyngbyabellin A with the literature values[3].

Position	Literature	Current	Differences
	δ_H (*J* in Hz)	δ_H (*J* in Hz)	H
**1**			
**2**			
**3**	5.31	5.32	-0.0019
**4**	1.33, 1.72	1.33, NO	0, NA
**5**	1.60	1.61	-0.0062
**6**	2.00, 2.22	NO, 2.23	NA. -0.0045
**7**			
**8**	2.05	2.06	-0.0049
**9**	1.31	1.33	-0.0153
**10**	1.36	1.37	-0.0074
**11**			
**12**			
**13**	8.09	8.11	-0.0025
**14**			
**15**	5.24	5.26	-0.0038
**15-NH**	7.27	NO	NA
**16**	1.97	NO	NA
**17**	1.13, 1.50	1.13, NO	0, NA
**18**	0.90	0.90	0
**19**	0.75	0.76	-0.0133
**20**			
**21**	3.70, 4.70	3.70, 4.71	0, -0.0021
**21-NH**	7.97	7.97	0
**22**			
**23**			
**24**	8.23	8.24	-0.0012
**25**			
**26**	6.13	6.14	-0.0016
**27**			
**28**	1.24	1.23	0.0081
**29**	1.38	1.39	-0.0072

[3] Luesch, H.; Yoshida, W.Y.; Moore, R.E.; Paul, V.J.; Mooberry, S.L. Isolation, structure determination, and biological activity of lyngbyabellin A from the marine cyanobacterium *Lyngbya majuscula*. *J. Nat. Prod*. **2000**, 63, 611-615. DOI: 10.1021/np990543q

NO: not observed. NA: not available

S4. Comparison of the ^{1}H chemical shifts of the isolated homohydroxydolabellin with the literature values[4].

	Literature	Current	Differences
**Position**	**δ_H (*J* in Hz)**	**δ_H (*J* in Hz)**	**H**
**1**			
**2**	2.96	2.97	-0.0034
**3**	5.40	5.41	-0.0019
**4**	1.76, 1.80	NO, 1.80	NA, 0
**5**	1.78		
**6**	2.17, 2.26	NO, NO	NA, NA
**7**			
**8**	2.12	2.13	-0.0047
**9**	1.25	1.26	-0.0080
**10**			
**11**			
**12**	8.18	8.18	0
**13**			
**14**	5.07	5.08	-0.0020
**15**	4.00, 4.04	NO, 4.03	NA, 0.0025
**16**			
**17**			
**18**	8.25	8.25	0
**19**			
**20**	6.26	6.26	0
**21**			
**22**	1.71, 1.78	NO, 1.80	NA, -0.0112
**23**	0.97	0.98	-0.0103
**24**	1.09	1.10	-0.0092
**25**	3.95	3.96	-0.0025

[4] Luesch, H.; Yoshida, W.Y.; Moore, R.E.; Paul, V.J. Structurally diverse new alkaloids from Palauan collections of the apratoxin-producing marine cyanobacterium *Lyngbya* sp. *Tetrahedron* **2002**, 58, 7959-7966. DOI: 10.1016/S0040-4020(02)00895-5

NO: not observed. NA: not available

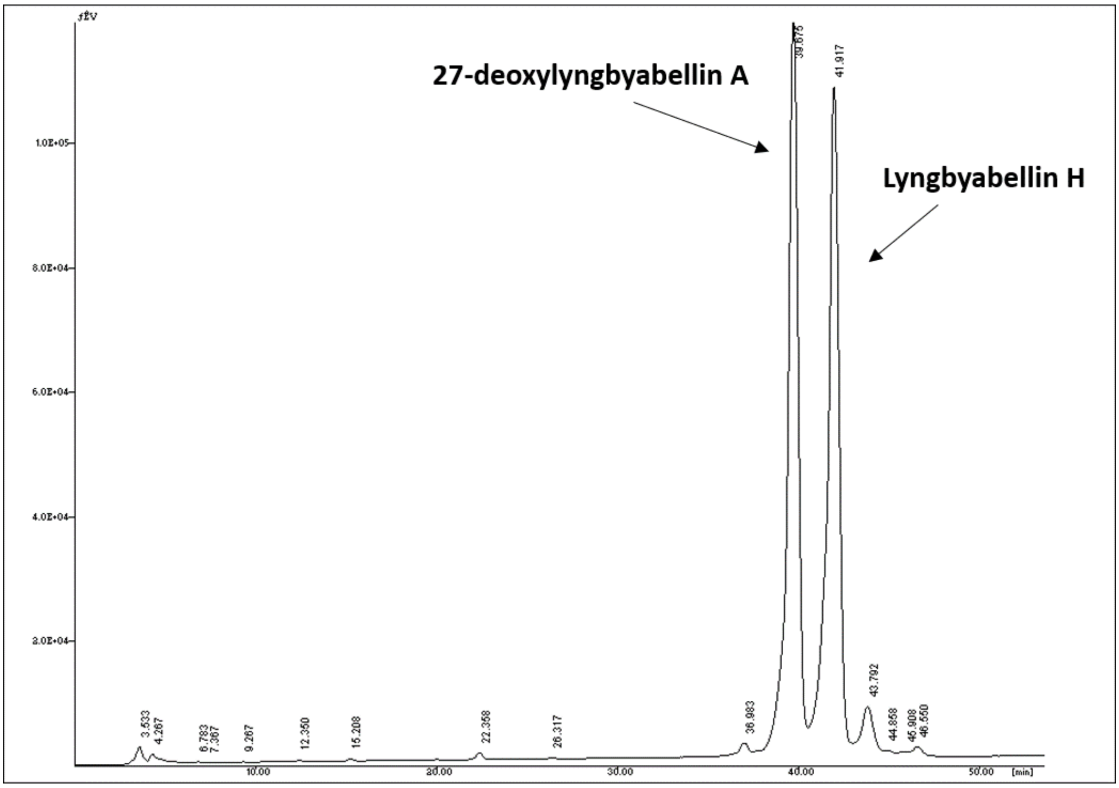

S5. RP-HPLC spectrum for the isolation of 27-deoxylyngbyabellin A and lyngbyabellin H.
Conditions: Cosmosil Cholester column (10 × 250 mm, 5 µm), gradient 0–60 min, 50%–70% MeCN, UV detection at 210 nm, flow rate of 3 mL/min.

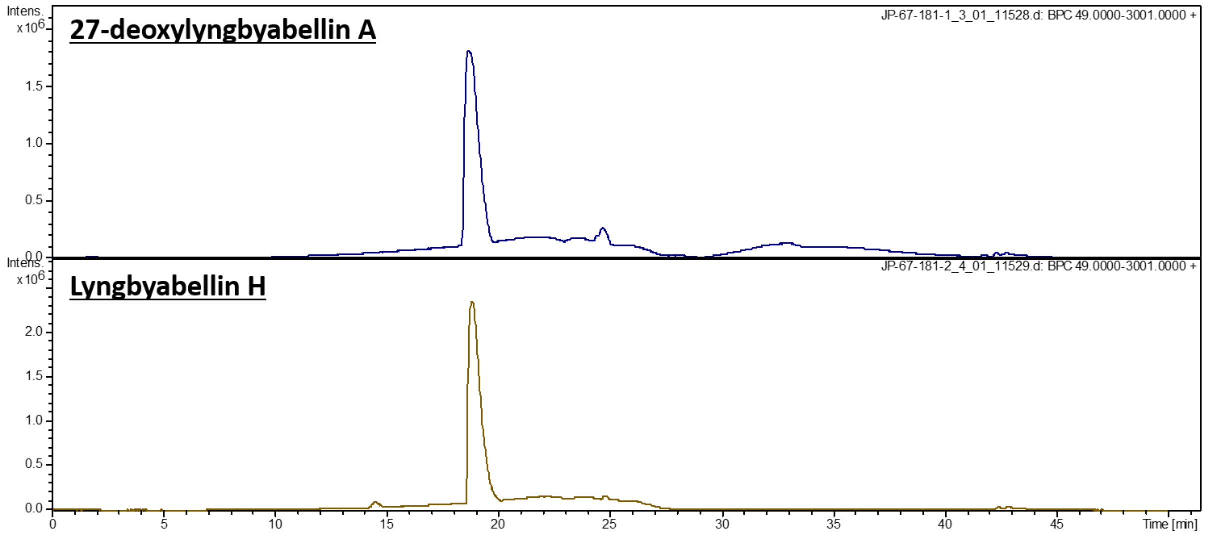

S6. LC/MS spectra for 27-deoxylyngbyabellin A and lyngbyabellin H.
Conditions: Cadenza CD-C18 column (2 × 150 mm, 3 µm), gradient: 0–20 min: 50%–100% MeCN + 0.1% FA (*v*/*v*) and 20–35 min: isocratic 100% MeCN + 0.1% FA (*v*/*v*), flow rate of 0.2 mL/min, 25 ℃.

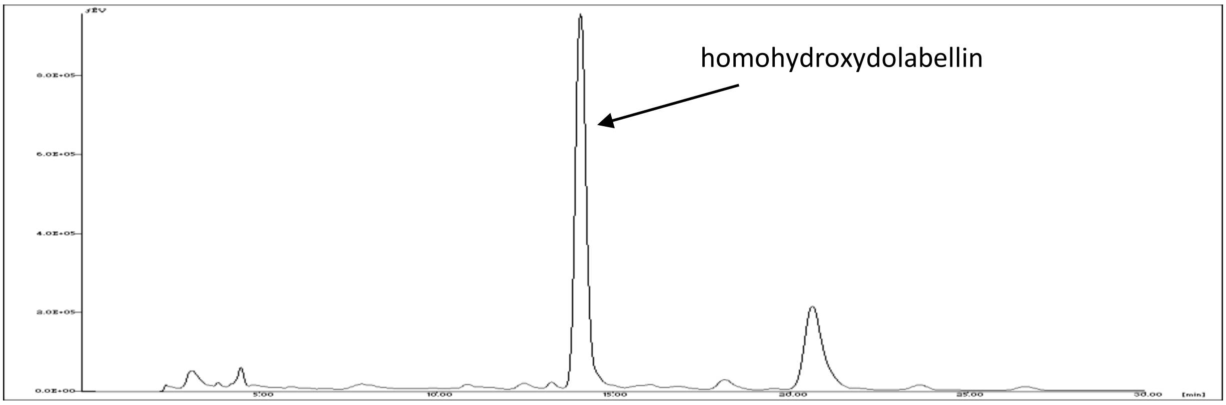

S7. RP-HPLC spectrum for the isolation of homohydroxydolabellin.
Conditions: Cosmosil Cholester column (4.6 × 250 mm, 5 μm), gradient 0–60 min, 50%–70% MeCN, UV detection at 210 nm, flow rate of 1 mL/min.

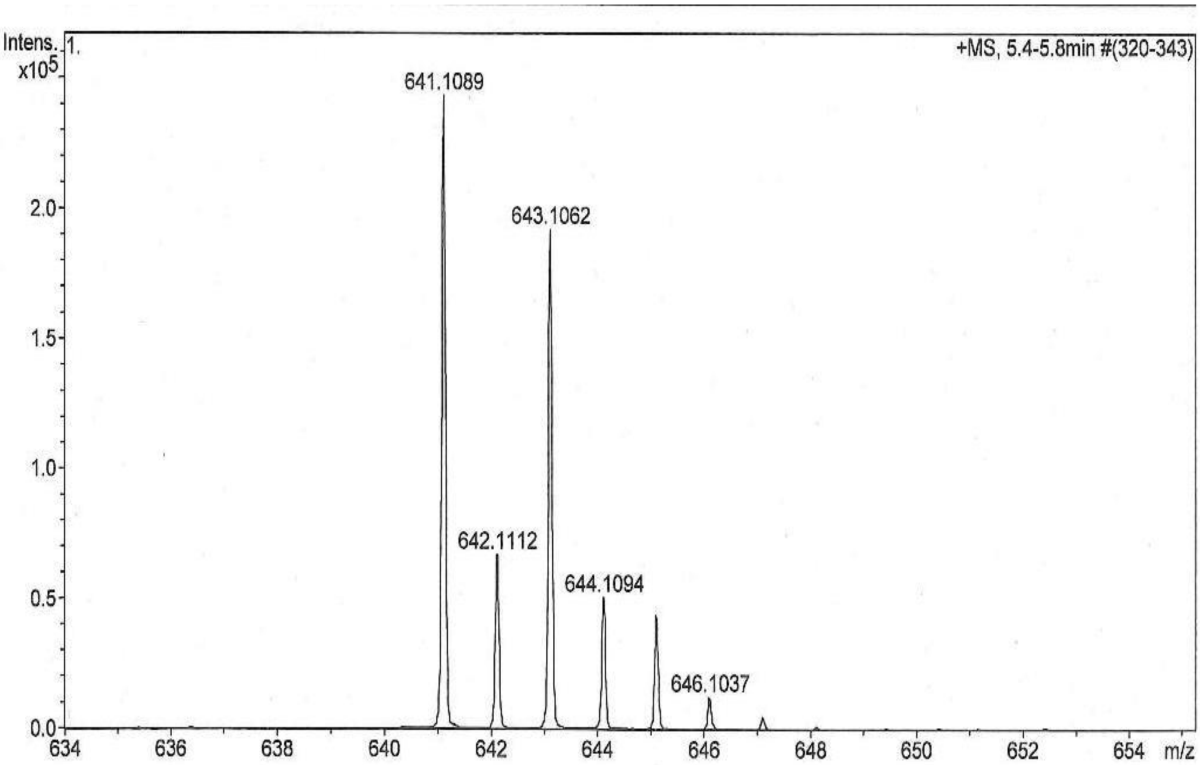

S8. LC/MS spectra for homohydroxydolabellin.
Conditions: Cadenza CD-C18 column (2 × 150 mm, 3 μm), gradient: 0–20 min: 50%–100% MeCN + 0.1% FA (*v*/*v*) and 20–35 min: isocratic 100% MeCN + 0.1% FA (*v*/*v*), flow rate of 0.2 mL/min, 25 ℃.
